# Homocysteine-Mediated Neuronal Pyroptosis Contributes to Brain Injury in Heatstroke Rats by Activating the m^6^A-YTHDF2-NLRP3 Pathway

**DOI:** 10.3390/cells14181437

**Published:** 2025-09-15

**Authors:** Shijia Zhang, Fang Xie, Xue Wang, Zhaowei Sun, Ling Zhang, Weiwei Liu, Xiaobing Chen, Lingjia Qian, Yun Zhao

**Affiliations:** 1 School of Basic Medical Sciences, Anhui Medical University, Hefei 230032, China; zsjdeyx2022@163.com (S.Z.); 18306826208@163.com (L.Z.); 2 Beijing Institute of Basic Medical Sciences, Beijing 100850, China; vancoxie@sina.com (F.X.); snowwang0326@foxmail.com (X.W.); sunzhw0820@163.com (Z.S.); lww168502@163.com (W.L.); 17859518569@163.com (X.C.)

**Keywords:** heat stroke, pyroptosis, prefrontal cortex, homocysteine, NLRP3, PC12 cell line

## Abstract

Heat stroke (HS) is a life-threatening condition that leads to neuronal injury, particularly in the prefrontal cortex, though its mechanisms remain unclear. In this study, we established a rat HS model and observed significant inflammatory responses and neuronal pyroptosis in the prefrontal cortex 6 h post-heat exposure, with the injury severity increasing over time. Mechanistically, HS activated the caspase-1/GSDMD-dependent pyroptosis pathway through NLRP3 inflammasome activation, resulting in IL-1β and IL-18 release. Additionally, HS caused a marked increase in homocysteine (Hcy) levels in both the serum and the prefrontal cortex, accompanied by reduced expression of the Hcy metabolic enzymes MTHFR and CSE, suggesting Hcy metabolism disruption. In vitro, Hcy induced pyroptosis in PC12 cells, elevating IL-1β, IL-18, and LDH levels. Notably, the NLRP3 inhibitor MCC950 mitigated this effect by reducing IL-18 and LDH release. Reducing Hcy in vivo alleviated neuronal pyroptosis and counteracted the YTHDF2-mediated decrease in NLRP3 mRNA m^6^A modification. Hcy reduced global m^6^A modification, YTHDF2 expression, and NLRP3 m^6^A modification in PC12 cells. This study reveals that the activation of a novel m^6^A-YTHDF2-NLRP3 pathway by Hcy underlies HS-induced neuronal injury, suggesting potential therapeutic targets for HS intervention.

## 1. Introduction

Heat stroke (HS) is a life-threatening condition characterized by a core body temperature exceeding 40 °C, accompanied by central nervous system dysfunction, such as delirium, seizures, or coma [1]. HS leads to multi-organ damage, with HS encephalopathy being its most prevalent and prominent manifestation. Recent multi-omics analyses have identified the cerebral cortex, particularly the anterior cingulate cortex (ACC), as the most severely affected region in HS [2].

HS involves multiple cell death modalities, with substantial evidence indicating its induction of apoptosis and autophagy [3,4]. In HS-induced hepatic injury, extracellular histones activate the NOD-like receptor family pyrin domain-containing 3 (NLRP3) inflammasome through a Toll-like receptor 9 (TLR9)-dependent signaling pathway, leading to hepatocyte pyroptosis [5]. Pyroptosis is a type of programmed cell death associated with inflammation marked by cell swelling, rupture of the plasma membrane, and the subsequent release of pro-inflammatory cellular components [6]. The NLRP3 inflammasome acts as the central molecular platform for pyroptosis, facilitating the release of interleukin-1 family cytokines (such as IL-1β and IL-18), the formation of ASC (apoptosis-associated speck-like protein containing a CARD) specks, and the activation of inflammatory caspases [6]. Importantly, NLRP3 inflammasome-driven neuronal pyroptosis has been linked to the development of several neurodegenerative diseases, including Alzheimer’s disease (AD), traumatic brain injury (TBI), and Parkinson’s disease (PD) [7]. However, the mechanisms underlying HS-induced neuronal pyroptosis remain poorly understood.

Homocysteine (Hcy), a sulfur-based non-protein amino acid, serves as an intermediate metabolite in methionine metabolism [8]. Hcy metabolism in the body primarily involves two pathways: the methylation pathway and the sulfur transfer pathway. In the methylation pathway, Hcy is converted into methionine through the action of 5,10-methylenetetrahydrofolate reductase (MTHFR), methionine synthase (MS), and betaine homocysteine methyltransferase (BHMT). In the sulfur transfer pathway, Hcy is ultimately metabolized through the action of cystathionine beta synthase (CBS) and cystathionine gamma lyase (CSE) [9]. The entire process of Hcy metabolism in the brain primarily relies on MS, MTHFR, CBS, and CSE [9]. Elevated Hcy levels can induce cellular oxidative stress, promote protein aggregation and dysfunction, stimulate apoptosis, and trigger inflammatory responses [10]. Clinical studies have reported that serum Hcy levels are significantly increased in patients with febrile seizures [11] while occupational heat exposure has also been shown to elevate plasma homocysteine concentrations [12]. Recent research has demonstrated that Hcy exacerbates macrophage pyroptosis by disrupting endoplasmic reticulum stress, endoplasmic reticulum–mitochondria coupling, and calcium homeostasis, thereby accelerating atherosclerosis progression [13].

N6-methyladenosine (m^6^A), the most common internal modification found in eukaryotic mRNA, regulates gene expression by modulating RNA metabolic processes including splicing, translation, and degradation [14,15]. Studies have demonstrated that m^6^A modification can ameliorate neural damage and suppress cell death (including pyroptosis and ferroptosis), with NLRP3 serving as a key molecular target [16]. The dynamic regulation of m^6^A involves three categories of proteins: methyltransferases (“writers”, e.g., the METTL3/14 complex; METTL14 is downregulated in traumatic brain injury [17]), demethylases (“erasers”, e.g., FTO/ALKBH5, which play crucial roles in cerebral ischemia [18]), and recognition proteins (“readers”). Among YTH family reader proteins, the cytoplasm-localized YTHDF2 regulates gene expression by promoting mRNA degradation, while the nucleus-localized YTHDC1 exhibits neuroprotective effects through its upregulation during early-stage ischemic stroke [19]. These findings suggest that the m^6^A modification system, particularly YTHDF2 and YTHDC1, may serve as potential therapeutic targets for central nervous system injuries. Hcy metabolism, which is part of the methionine cycle, plays a pivotal role in maintaining methylation homeostasis. Within this cycle, S-adenosylmethionine (SAM) acts as a methyl donor to regulate m^6^A levels and influence gene expression [20]. Recent research has indicated [21] that in Hcy-induced cognitive dysfunction, betaine treatment enhances the m^6^A-YTHDF2-dependent mechanism, reduces NLRP3 mRNA stability, and inhibits the pyroptosis pathway, thereby exerting neuroprotective effects.

These findings suggest that Hcy may play a critical role in HS-induced cerebral injury and cellular pyroptosis. In this context, the present study investigates the involvement and underlying mechanisms of Hcy in the pyroptosis of prefrontal cortical neurons in a HS rat model.

## 2. Materials and Methods

### 2.1. Rat HS Model

Specific pathogen-free (SPF) adult male Wistar rats (8 weeks old, 200–250 g) were obtained from Beijing SPF Biotechnology Co., Ltd. (Beijing, China)The animals were housed in SPF-grade facilities at the Academy of Military Medical Sciences under controlled conditions: ambient temperature of 24 ± 2 °C, humidity of 50 ± 10%, and a 12-h light/dark cycle. Following a 5-day acclimatization period with ad libitum access to food and water, the rats were randomly allocated into three experimental groups, with 12 rats in each group. The animal experiments and protocols received approval from the Institutional Animal Care and Ethics Committee of the Academy of Military Medicine Sciences.

Rats in the immediate HS group (HS0) were placed in a preheated artificial climate chamber (39.5 ± 0.5 °C, 65 ± 5% humidity) until their core body temperature reached 42 °C, at which point, heatstroke was considered successfully induced, and tissue collection and subsequent experiments were immediately carried out. Rats in the 6-h post-HS group (HS6) were subjected to the identical HS induction protocol as the HS0 group, and then moved from the climate chamber to their home cages immediately after the onset of HS, where they were allowed to recover for 6 h under standard conditions (24 ± 2 °C) before tissue collection and subsequent experiments. All heat exposures were initiated at 08:00 daily. During heat exposure, the animals were fasted with water restriction. The core body temperature was monitored every 30 min using a rectal thermometer (Taiwan, China), with the measurement frequency increased to every 10 min once the rectal temperature reached 41.5 °C. The control rats underwent the same procedure without being heated.

### 2.2. Vitamin B-Complex Treatment

To determine the role of Hcy in HS, a vitamin B complex was used to lower Hcy levels, which consisted of folic acid (10 mg/kg/d), vitamin B6 (24 mg/kg/d), vitamin B12 (20 μg/kg/d), and betaine (10 mg/kg/d) (Michigan, USA). The corresponding control group was administered the solvent via gavage. All groups received daily gavage administration at 9:00 AM (2 mL volume) for 2 weeks. Before inducing heat stress (HS) in the rats, the HS6 + VitB group received a pre-treatment with a composite B vitamin oral gavage, while the control group and HS6 + H_2_O group were administered H_2_O instead of the composite B vitamins.

### 2.3. Transmission Electron Microscopy (TEM)

The rat was euthanized via intraperitoneal anesthesia administration (2,2,2-tribromoethanol and tert-amyl alcohol (0.2 mL/10 g body weight)), and prefrontal cortex tissue samples (1 mm^3^) were immediately excised and fixed in pre-chilled (4 °C) electron microscopy-grade fixative (Wuhan, China). The fixed specimens underwent standard processing including dehydration, resin embedding, and ultrathin sectioning using a diamond knife-equipped ultramicrotome (Weztlar, Germany). Then, the morphology of neurons was observed using a transmission electron microscope (Massachusetts, USA).

### 2.4. Quantitative Real-Time PCR (qRT-PCR)

Total RNA was isolated using the TRIzol reagent (#94289, Michigan, USA). The RNA concentration was determined spectrophotometrically (Massachusetts, USA). For cDNA synthesis, 1000 ng of total RNA was reverse transcribed using a commercial reverse transcription kit (#G490, Houston, Canada). qRT-PCR was performed using TB Green Premix Ex Taq II (#DRR820A, Kusatsu, Japan) and a LightCycler 480 system (Basel, Switzerland).

Gene-specific primers (Table 1) were designed using the Primer-BLAST tool (National Center for Biotechnology Information). The 10 µL PCR reaction mixture contained 1.0 µL of the cDNA template, 0.2 µL each of the forward and reverse primers (10 µM), 5.0 µL of TB Green Premix Ex Taq II (2×), and 3.6 µL of nuclease-free water. Amplification was performed under the following conditions: initial denaturation at 95 °C for 2 min; 50 cycles of denaturation at 95 °C for 10 s, annealing at 60 °C for 10 s, and extension at 72 °C for 15 s. All reactions were performed in technical triplicates, with melt curve analysis conducted to verify amplification specificity. Relative gene expression was quantified using the comparative threshold cycle (2−ΔΔCq) method.

### 2.5. Enzyme-Linked Immunosorbent Assay (ELISA)

The following commercial ELISA kits were used to measure the concentrations of caspase-1, caspase-4, SAM, and SAH in the rat prefrontal cortex samples, as well as IL-18, IL-1β, LDH, SAM, and SAH concentrations in the rat serum and cell culture supernatants, including the Caspase-1 Activity Assay Kit (#KTA3020, Wuhan, China), Caspase-4 Activity Assay Kit (#KTA3032, Wuhan, China), Rat IL-18 ELISA Kit (#KTE3044, Wuhan, China), Rat IL-1β ELISA Kit (#KTE9001, Wuhan, China), Rat L-Lactate Dehydrogenase ELISA Kit (#AKC0003M, Shanghai, China), SAM ELISA Kit (A112998, Shanghai, China), and SAM ELISA Kit (A113059, Fusheng, China). A syringe needle (Foshan, China) was inserted from the apex towards the ventricle to collect blood into a serum separator tube (Shijiazhuang, China). The tube was inverted and mixed several times, and then centrifuged at 3000 rpm for 10 min. The supernatant was collected and served as a serum sample.

### 2.6. Western Blotting

The prefrontal cortex tissues were homogenized in RIPA buffer (#R0020, Beijing, China) and centrifuged. The protein concentrations in the supernatants were determined using a BCA Protein Assay Kit (#W9924, Beijing, China). Equal amounts of protein were separated by SDS-PAGE and transferred onto PVDF membranes (Hercules, CA, USA). After blocking with 5% non-fat milk for 1 h at room temperature, the membranes were incubated overnight at 4 °C with primary antibodies (all diluted in TBST) including NLRP3 (Danvers, MA, USA; 1:1000); caspase-4, IL-18, ASC, GSDMD, IL-1β, caspase-1, CSE, MS, MTHFR, CBS (Wuhan, China; 1:1000); and β-actin (Wuhan, China; 1:5000) antibodies. Following TBST washes, the membranes were incubated with horseradish peroxidase-conjugated secondary antibodies (goat anti-rabbit IgG (H + L) (#RS0002, California, USA; 1:2000) or goat anti-mouse IgG (H + L) (#RS0001, California, USA; 1:2000) for 2 h at room temperature. The protein bands were visualized using a chemiluminescence detection system (Wuhan, China).

### 2.7. Immunofluorescence

The brains were fixed (2–3 h), dehydrated in 10%/20%/30% sucrose, embedded in OCT (Kunshan, China), and frozen. Prefrontal cortex sections were cryosectioned (Weztlar, Germany), mounted on chilled slides (Hefei, China), dried (37 °C), washed in PBS (15 min), permeabilized (0.3% Triton X-100, 30 min), and rinsed (3× PBS). Antigen retrieval was performed using a 1× sodium citrate buffer (10 min, heated), followed by PBS washes (3×). The sections were blocked (4 °C, 1–2 h), and then incubated with primary antibodies (200 µL/slide, 4 °C, 24 h): mouse anti-NeuN (#ab104224, Cambridge, MA, USA; 1:100), rabbit anti-NLRP3 (#27458-1-AP, Wuhan, China; 1:100), and rabbit anti-GSDMD (#20770-1-AP, Wuhan, China; 1:100). After PBS washes (3×), secondary antibodies (200 µL/slide) were applied: goat anti-rabbit IgG H&L (#ab150080, Cambridge, MA, USA; 1:200), goat anti-mouse IgG H&L (#ab150077, Cambridge, MA, USA; 1:200, 2 h, room temperature in the dark). Following PBS washes and drying, the sections were mounted with DAPI-containing mounting medium (#F6057, Michigan, USA) and coverslipped for confocal microscopy (Hong Kong, China) observation. Using ImageJ 1.8.0 software, quantitative analysis of the fluorescence signals was performed using the thresholding method with area analysis.

### 2.8. Cell Culture and Intervention

The PC12 cell line (highly differentiated rat adrenal pheochromocytoma cells) (#CL-0481, Wuhan, China) was used as a neuronal model and cultured in RPMI-1640 basal medium supplemented with 10% FBS and 1% penicillin/streptomycin. The cells were maintained at 37 °C in a 5% CO_2_ incubator.

A 50 µM homocysteine solution (#H4628, Michigan, USA) was prepared using distilled deionized water (ddH_2_O). Cells exhibiting optimal growth conditions were digested, resuspended, and subsequently seeded into new culture dishes. After growing them for 6–8 h to allow for cell attachment, the cell morphology was observed under a microscope. The medium was then discarded and replaced with fresh medium containing 50 μM Hcy for the Hcy-treated group, 10 μM MCC950 (#HY-12815, New Jersey, USA) for the MCC950 group, or 10 μM MCC950, followed by a 4-h pretreatment, after which, 50 μM Hcy was added to the MCC950 + Hcy group. All cell cultures were maintained in a 37 °C incubator with 5% CO_2_ for 24 h.

### 2.9. m^6^A Dot Blot Assay

The experiments were performed according to previously reported methods [22]. mRNA samples with a concentration of 150 ng/μL were denatured via heating at 95 °C for 3 min. Subsequently, 2 μL of the denatured mRNA samples were spotted onto an N+ Nylon Transfer Membrane (#TM-NY-S-45, Beijing, China). After UV irradiation (30 min), the membranes were rinsed with TBST (3 min) and blocked with 5% skim milk (90 min). They were then incubated overnight at 4 °C with rabbit anti-m^6^A antibody (#202003, California, USA, 1:1000). Then, the membranes were incubated with goat anti-rabbit IgG (H + L) (#RS0002, California, USA; 1:2000) for 2 h at room temperature. The blots were visualized using enhanced chemiluminescence. Post-imaging, the membranes were stained with 0.2% methylene blue (10 min), rinsed with water (10 min), air-dried, and documented.

### 2.10. Methylated RNA Immunoprecipitation (MeRIP) qPCR Assay

This study performed MeRIP experiments using the m^6^A MeRIP kit (#p901824, New York, NY, USA), with the RNA samples (10 μg) prepared according to the manufacturer’s protocol. After immunoprecipitation, the enrichment of mRNA with m^6^A modifications was assessed using qRT-PCR.

### 2.11. Statistical Analysis

All statistical analyses were conducted using GraphPad Prism 9.0 software. A two-tailed paired *t*-test was employed to compare the means between two groups, while one-way ANOVA with Tukey’s post hoc test was used for multiple group comparisons. The data are presented as the mean ± SEM, and a *p*-value of <0.05 was considered statistically significant.

## 3. Results

### 3.1. Increased Neuronal Pyroptosis in Prefrontal Cortex of HS Rats

**Figure 1:**
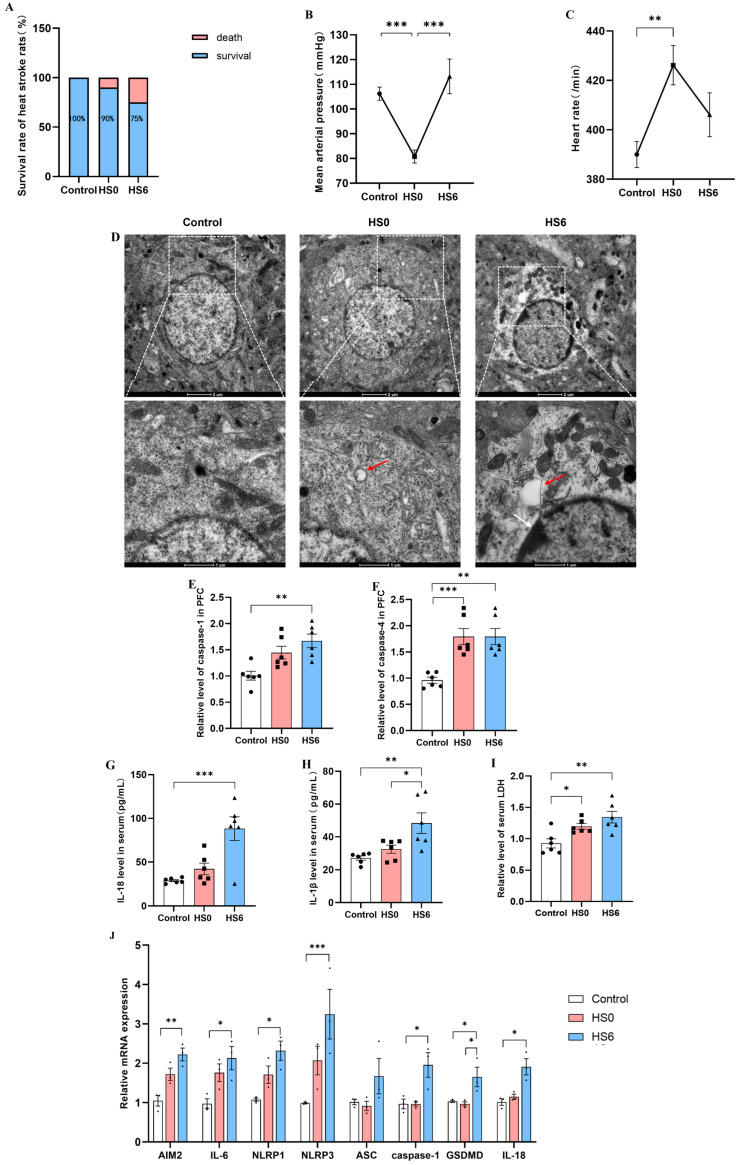
Effects of heatstroke (HS) on neuronal pyroptosis in rat prefrontal cortex. (**A**–**C**) Survival rate, mean arterial pressure, and heart rate of control group, HS0 group, and HS6 group rats (*n* = 12). (**D**) Representative transmission electron microscopy images of control, HS0, and HS6 groups. Scale bar: 2 μm. Red arrows indicate vesicular structures; white arrows show increased heterochromatin condensation and aggregation. (**E**,**F**) ELISA analysis of caspase-1 and caspase-4 levels in prefrontal cortex (*n* = 6). (**G**–**I**) ELISA analysis of IL-18, IL-1β, and LDH levels in serum (*n* = 6). (**J**) mRNA expression changes of pyroptosis-related genes in rat prefrontal cortex (*n* = 3). * *p* < 0.05, ** *p* < 0.01, *** *p* < 0.001.

The survival rate of the HS0 rats was 90%, and it decreased to 75% in the HS6 rats (Figure 1A). Successful model establishment was confirmed by the immediate decrease in mean arterial pressure and increase in heart rate in the HS0 rats, followed by partial recovery after 6 h (Figure 1B,C). Cellular swelling and membrane rupture represent hallmark features of pyroptosis. To investigate the occurrence of neuronal pyroptosis in the prefrontal cortex after HS, we examined ultrastructural changes using TEM. Compared with controls, the HS0 group exhibited vesicular structures near the cytoplasm and plasma membrane with reduced organelles. The HS6 group demonstrated additional pathological alterations including membrane rupture, increased heterochromatin condensation, and irregular chromatin aggregation along the nuclear envelope (Figure 1D). ELISA analysis revealed that the levels of caspase-1 and caspase-4 significantly increased in prefrontal cortex of the HS6 rat (Figure 1E,F). Meanwhile, the levels of IL-18, IL-1β and LDH were elevated in the serum of the HS6 rats (Figure 1G,I). qRT-PCR analysis showed that the mRNA levels of pyroptosis-related genes were markedly upregulated in the prefrontal cortex of the HS6 group, including *AIM2, IL-6, NLRP1, NLRP3, caspase-1, GSDMD,* and *IL-18* (Figure 1J). These findings collectively demonstrated the occurrence of neuronal pyroptosis in the prefrontal cortex at 6 h post-HS onset.

### 3.2. Pyroptosis in Prefrontal Cortical Neurons of HS Rats Primarily Occurs Through the Classical Pathway

**Figure 2:**
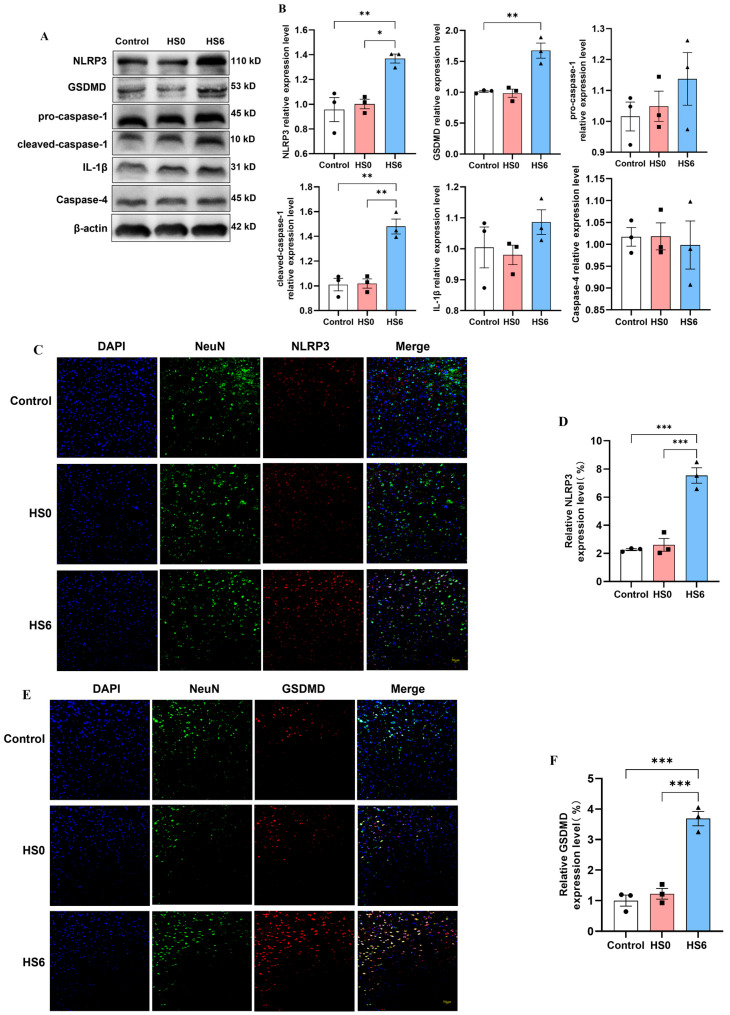
Effects of HS on the classical pyroptosis pathway in rat prefrontal cortical neurons. (**A**) Expression levels of pyroptosis-related proteins in prefrontal cortex across experimental groups (*n* = 3). (**B**) Quantitative analysis of pyroptosis-related protein expression in prefrontal cortex (*n* = 3). (**C**,**D**) Immunofluorescence co-localization and quantitative analysis of NLRP3 in neurons in prefrontal cortex (*n* = 3). (**E**,**F**) Immunofluorescence co-localization and quantitative analysis of GSDMD in neurons in prefrontal cortex (*n* = 3). * *p* < 0.05, ** *p* < 0.01, *** *p* < 0.001.

Western blot analysis showed significant upregulation of classical pyroptosis pathway components in the HS6 group compared to the controls, including NLRP3, GSDMD, and cleaved caspase-1 (Figure 2A,B). While IL-1β and pro-caspase-1 showed increasing trends, the non-classical pathway marker caspase-4 remained unchanged. These findings confirm enhanced pyroptosis at 6 h post-HS and suggest that the classical pathway is predominantly involved. To verify neuronal-specific activation of this pathway, we performed immunofluorescence co-staining of neurons for NLRP3 and GSDMD. The HS6 group exhibited markedly increased expression of both NLRP3 and GSDMD in prefrontal cortical neurons (Figure 2C,F), demonstrating classical pyroptosis pathway activation in neurons following HS.

### 3.3. Hcy Metabolism Disorder in the Prefrontal Cortex of HS Rats

**Figure 3:**
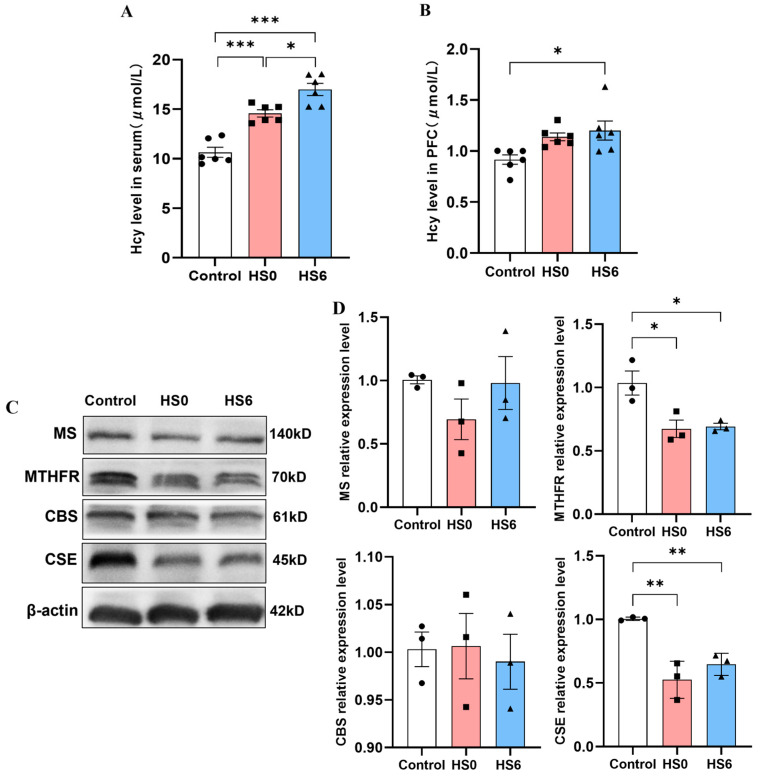
Effects of HS on homocysteine (Hcy) metabolism in rats. (**A**) ELISA analysis of Hcy levels in serum (*n* = 6). (**B**) ELISA analysis of Hcy levels in prefrontal cortex (*n* = 6). (**C**,**D**) Protein expression levels and quantitative analysis of Hcy metabolic enzymes in prefrontal cortex (*n* = 3). * *p* < 0.05, ** *p* < 0.01, *** *p* < 0.001.

Given that Hcy can induce oxidative stress, cellular dysfunction, cell death, and inflammation, we investigated its potential role in HS pathogenesis and neuronal pyroptosis. The results showed that Hcy levels were significantly increased in the serum of both the HS0 and HS6 rats, and in the prefrontal cortex of the HS6 group compared to the controls (Figure 3A,B). The Western blot results showed that the Hcy metabolic enzymes MTHFR and CSE were markedly downregulated in the prefrontal cortex of the HS rats (Figure 3C,D). These findings indicated that HS induces cerebral Hcy accumulation accompanied by impaired Hcy metabolism.

### 3.4. Hcy Mediates Pyroptosis of Neurons in the Prefrontal Cortex of HS Rats

**Figure 4:**
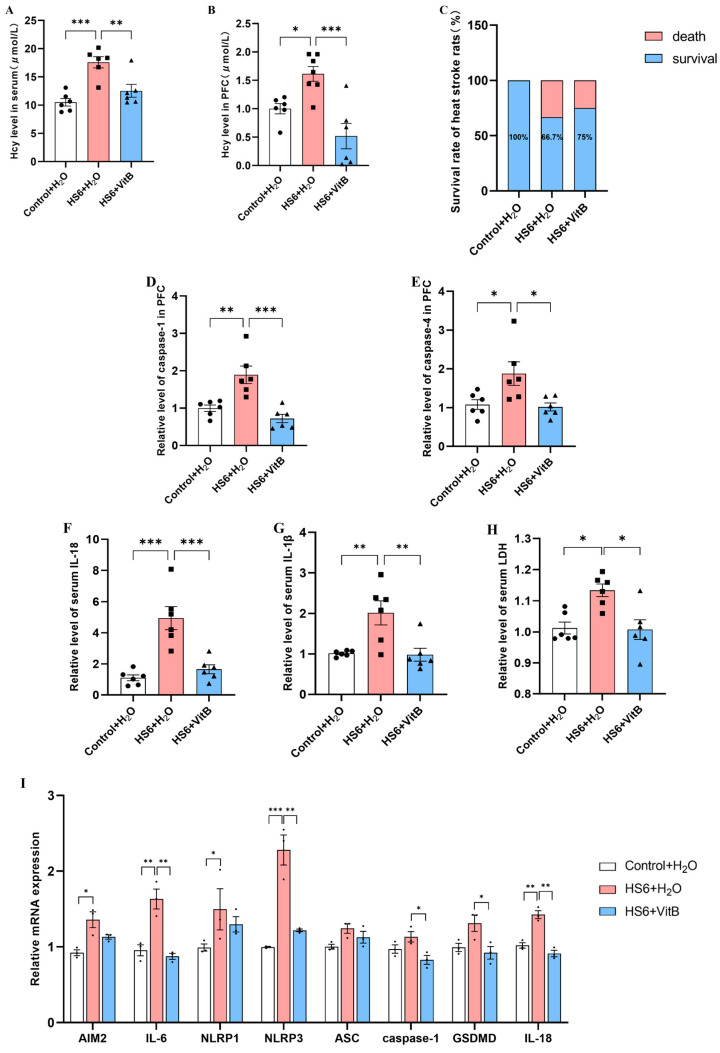
Hcy-mediated neuronal pyroptosis in HS-induced brain injury. (**A**) ELISA analysis of Hcy levels in serum (*n* = 6). (**B**) ELISA analysis of Hcy levels in prefrontal cortex (*n* = 6). (**C**) Survival rate of rats (*n* = 12). (**D**,**E**) ELISA analysis of caspase-1 and caspase-4 levels in prefrontal cortex (*n* = 6). (**F**–**H**) ELISA analysis of IL-18, IL-1β, and LDH levels in serum (*n* = 6). (**I**) mRNA expression changes of pyroptosis-related genes in rat prefrontal cortex (*n* = 3). * *p* < 0.05, ** *p* < 0.01, *** *p* < 0.001.

To investigate whether Hcy contributes to pyroptosis in HS rat neurons in the prefrontal cortex, we administered a vitamin B complex (VitB) to downregulate Hcy levels in the HS rats. ELISA was performed to measure pyroptosis-related proteases and inflammatory factors, while qRT-PCR was used to assess pyroptosis-related gene expression. Compared to the HS6 + H_2_O group, VitB administration significantly decreased Hcy levels in both the serum and prefrontal cortex of the HS rats, with an improved 6-h survival rate in the HS rats (Figure 4A–C). ELISA showed that Hcy reduction markedly lowered the caspase-1 and caspase-4 levels in the prefrontal cortex, along with decreased serum IL-18, IL-1β, and LDH levels (Figure 4D–H). The qRT-PCR analysis showed downregulated expression of *IL-6, NLRP3, caspase-1, GSDMD,* and *IL-18* in the prefrontal cortex following Hcy reduction (Figure 4I). These findings suggest that lowering Hcy levels reduces prefrontal cortical pyroptosis in HS rats.

### 3.5. NLRP3 Inhibition Attenuates Hcy-Induced Pyroptosis in PC12 Cells

**Figure 5:**
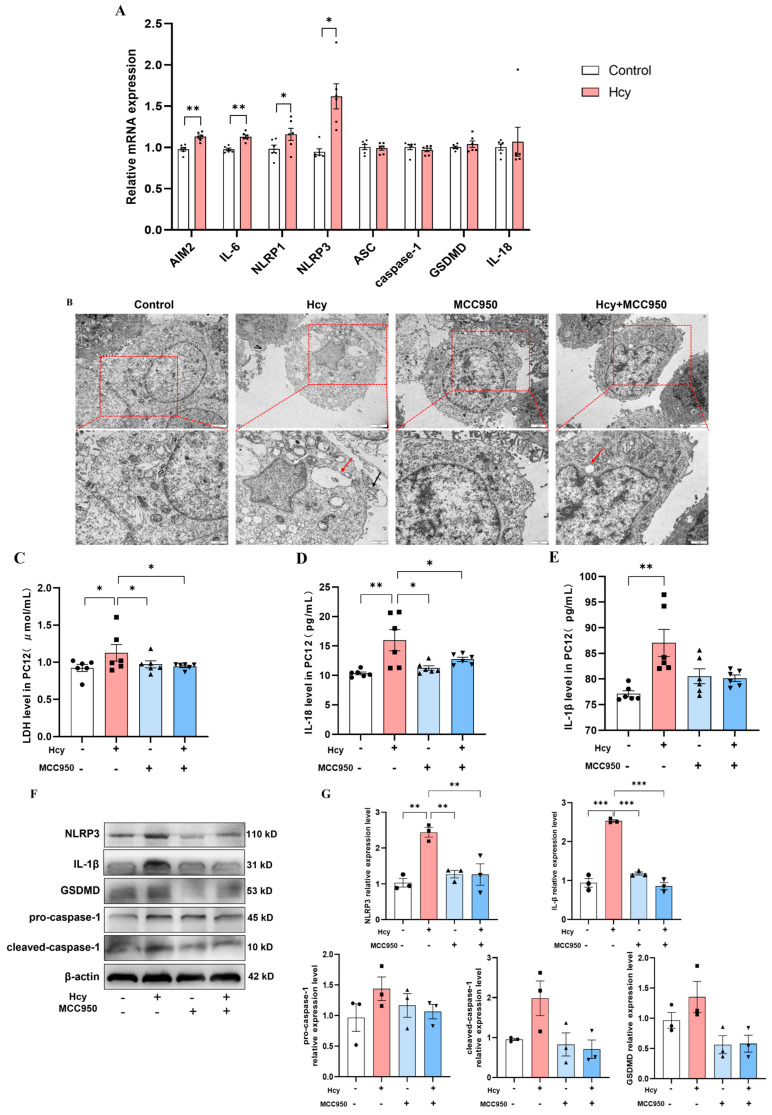
Role of NLRP3 in Hcy-induced pyroptosis in PC12 cells. (**A**) mRNA expression changes of pyroptosis-related genes in PC12 cells (*n* = 6). (**B**) Morphological features of neuronal pyroptosis in control, Hcy, MCC950, and Hcy + MCC950 groups. Scale bar: 2 μm. Red arrows indicate vacuolar structures; white arrows show nuclear membrane invagination; black arrows point to mitochondrial cristae disruption. (**C**–**E**) ELISA analysis of LDH, IL-18, and IL-1β levels in cell culture supernatants (*n* = 6). (**F**,**G**) Protein expression levels and quantitative analysis of pyroptosis-related proteins in PC12 cells (*n* = 3). * *p* < 0.05, ** *p* < 0.01, *** *p* < 0.001.

The PC12 cell line was utilized to investigate Hcy-induced pyroptosis. After a 24-h treatment with 50 μM Hcy, a significant upregulation of *AIM2*, *IL-6*, *NLRP1*, and *NLRP3* was observed (Figure 5A), indicating the activation of pyroptosis. To examine NLRP3′s role, we used MCC950 for specific NLRP3 inhibition. TEM revealed the characteristic pyroptotic morphology in the Hcy-treated cells, including nuclear membrane invagination, cytoplasmic vacuolization, and membrane rupture with cytoplasmic leakage. These ultrastructural changes were rarely observed in the cells pretreated with MCC950 and exposed to the Hcy intervention (Figure 5B). ELISA demonstrated Hcy-induced elevations in LDH, IL-18, and IL-1β, with MCC950 normalizing the LDH and IL-18 levels (Figure 5C–E). Western blot confirmed decreased NLRP3 and IL-1β protein expression with MCC950 treatment (Figure 5F,G). Collectively, these findings demonstrate that NLRP3 inhibition mitigates Hcy-induced pyroptosis in PC12 cells.

### 3.6. Hcy Mediates NLRP3 m^6^A Modification and YTHDF2 Expression in the Prefrontal Cortex of HS Rats

**Figure 6:**
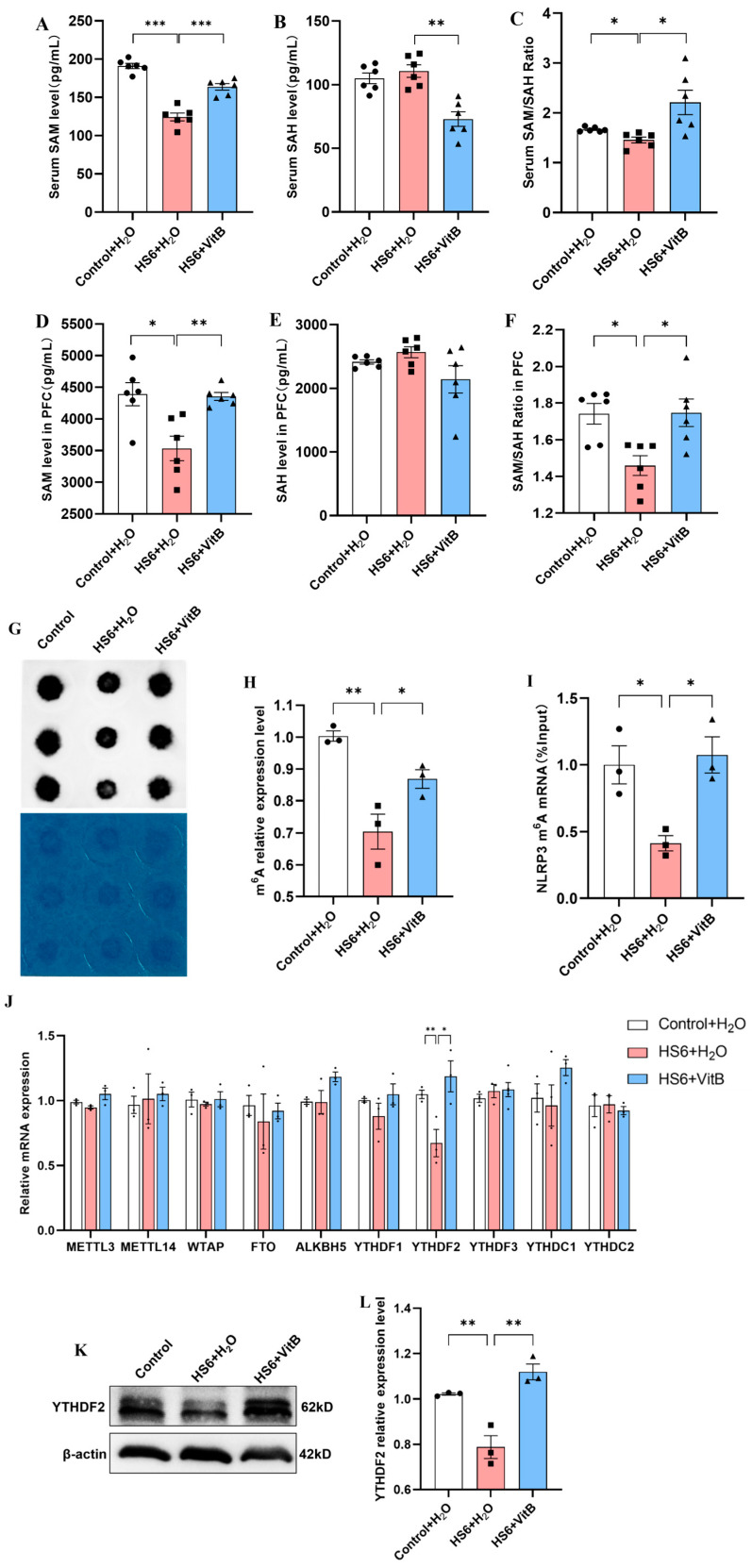
Effect of Hcy on NLRP3 m^6^A levels in HS rats. (**A**–**C**) Rat serum SAM and SAH levels and SAM/SAH ratio (*n* = 6). (**D**–**F**) SAM and SAH levels and SAM/SAH ratio in the prefrontal cortex of rats (*n* = 6). (**G**) mRNA m^6^A level in the prefrontal cortex of rats (*n* = 3). (**H**) Statistical analysis of mRNA m^6^A levels in the prefrontal cortex of rats (*n* = 3). (**I**) m^6^A level of NLRP3 mRNA in the prefrontal cortex of rats (*n* = 3). (**J**) Levels of m^6^A modification of related genes (*n* = 3). (**K**,**L**) YTHDF2 protein expression results (*n* = 3). * *p* < 0.05, ** *p* < 0.01, *** *p* < 0.001.

The ELISAs and dot blot assays showed that the HS6 + H_2_O group exhibited significantly reduced SAM levels, methylation capacity index, and global m^6^A levels in both the serum and prefrontal cortex, which were restored following Hcy downregulation (Figure 6A–H). MeRIP-qPCR showed that the m^6^A level of NLRP3 mRNA in the prefrontal cortex of the HS6 + H_2_O rats was significantly reduced, but it returned to normal levels after the VitB intervention (Figure 6I). qRT-PCR screening of ten genes associated with the regulation of m^6^A methylation enzymes revealed a significant downregulation in the expression of *YTHDF2* (an m^6^A “reader” protein) in the HS6 + H_2_O group, which was subsequently restored following VitB treatment (Figure 6J). Western blot analysis showed a significant reduction in the expression of YTHDF2 protein in the HS6 + H_2_O group compared to the Control + H_2_O group, with its expression level restored to normal levels following VitB treatment (Figure 6K,L). These results suggest that Hcy mediates HS-induced neuronal pyroptosis in the rat prefrontal cortex by regulating NLRP3 m^6^A modification and YTHDF2 expression.

### 3.7. Hcy Impairs m^6^A Modification and YTHDF2 Expression in PC12 Cells

**Figure 7:**
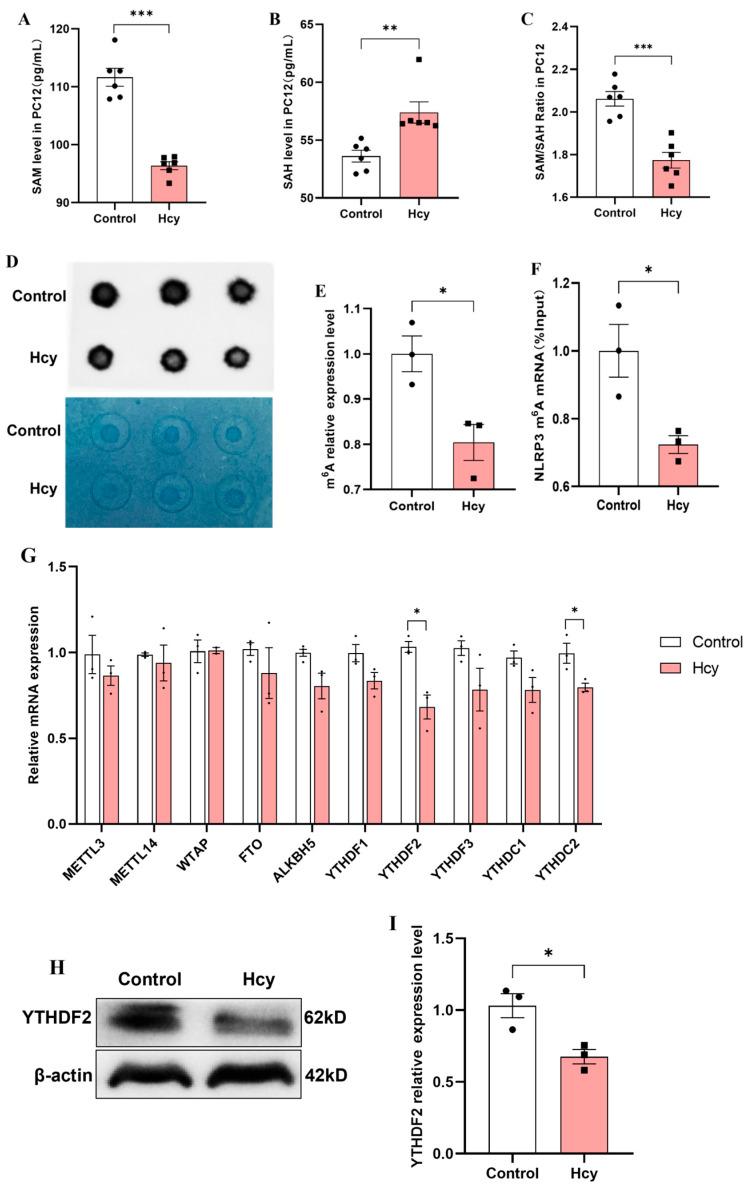
Effect of Hcy on mRNA m^6^A methylation modification in PC12 cells. (**A**–**C**) SAM and SAH levels and SAM/SAH ratio of PC12 cells (*n* = 6). (**D**,**E**) mRNA m^6^A level in PC12 cells (*n* = 3). (**F**) Effect of Hcy on NLRP3 m^6^A level in PC12 cells (*n* = 3). (**G**) Expression levels of m^6^A methylation regulatory factor in PC12 cells (*n* = 3). (**H**,**I**) Expression level of YTHDF2 protein in PC12 cells (*n* = 3). * *p* < 0.05, ** *p* < 0.01, *** *p* < 0.001.

To further verify the effect of Hcy on pyroptosis in PC12 cells, we examined its impact on mRNA m^6^A levels in PC12 cells. The results showed that compared with the control group, Hcy significantly decreased SAM levels and the SAM/SAH ratio and elevated SAH levels (Figure 7A–C). Dot blot analysis showed a marked reduction in global m^6^A modification in Hcy-treated cells (Figure 7D,E). MeRIP-qPCR further showed that Hcy significantly diminished NLRP3 mRNA m^6^A methylation (Figure 7F), indicating that elevated Hcy levels impair both global and NLRP3-specific m^6^A modification in PC12 cells. Additionally, we assessed the expression of m^6^A regulatory proteins in PC12 cells following Hcy treatment. qRT-PCR analysis indicated that Hcy significantly downregulated *YTHDF2* and *YTHDC2* mRNA levels (Figure 7G). Western blotting showed that YTHDF2 protein expression was also significantly reduced in Hcy-treated PC12 cells (Figure 7H,I). These findings collectively suggest that elevated Hcy levels suppress YTHDF2 expression in PC12 cells, mirroring the observations in rat prefrontal cortex tissues.

## 4. Discussion

As global warming progresses, HS is gradually becoming an important health issue [23,24], making it a critical public health concern. Encephalopathy represents the earliest pathological manifestation of HS, which is marked by neurological symptoms such as altered mental status, delirium, and coma, and precedes dysfunction in other organs [25]. Clinical data has indicated a 57% one-year survival rate for HS, with many survivors experiencing persistent neurological deficits [26]. Approximately 20% of patients developed long-term neurological impairments [27], which are frequently misdiagnosed and results in high mortality rates. While research has predominantly focused on hippocampal damage [28], the prefrontal cortex, which is essential for motor coordination, remains understudied [29]. The hypothalamus, especially the preoptic area (PO/AH), serves as the primary thermoregulatory center, though studies have also implicated the prefrontal cortex and limbic regions in thermoregulation [30]. HS induces widespread central nervous system damage, including in the cerebellum, hippocampus, basal ganglia, and cerebral cortex [31]. Recent studies have shown that despite progress in understanding HS-induced cell death mechanisms in several tissues, the neurological effects, particularly pyroptosis in prefrontal cortex neurons, are less explored. We observed significant neuronal damage in the prefrontal cortex of rats at 6 h post-heat exposure, including pronounced inflammatory responses, organelle pathology, and enhanced pyroptosis compared to the immediate post-heat exposure and control groups. The time-dependent progression of pyroptosis, which is consistent with previous reports [32,33,34], explains the persistent mortality risk despite cooling interventions [35,36]. Overall, the above findings highlight a critical 6-h therapeutic window for targeting pyroptosis and inflammation in clinical management.

Pyroptosis proceeds through three distinct pathways: the classical and non-canonical pathways, and a newly identified caspase-3/8-mediated pathway [37]. It involves five major inflammasomes: NLRP3, AIM2, NLRP1, PYRIN, and NLRC4. NLRP receptors play a key role in pyroptosis, with the NLRP3 inflammasome being the most widely studied. NLRP1 is another known NLRP member associated with pyroptosis. It activates the inflammasome and induces caspase-1 activation by sensing various endogenous and exogenous stimuli, such as bacterial toxins [38]. In the canonical pyroptosis pathway, these inflammasomes activate caspase-1 and cleave GSDMD, releasing inflammatory factors, triggering inflammatory responses, and inducing pyroptosis [39]. The non-canonical pathway employs caspase-11/4/5, which directly recognize bacterial lipopolysaccharide (LPS), triggering pyroptosis independently of inflammasomes. The newly described pathway activates caspase-3/8 to cleave gasdermin E (GSDME), resulting in an incomplete form of pyroptosis [40]. Building on the previous finding that HS increases pyroptosis in rat prefrontal cortical neurons, this study observed elevated caspase-1 and caspase-4 levels using ELISAs, while the Western blot analysis showed no change in caspase-4 expression. These findings suggest that caspase-4 activation may occur via post-translational modifications or rapid degradation upon inflammasome stimulation rather than transcriptional upregulation, consistent with studies indicating that caspase-4 is cleaved and activated without alterations in total protein levels in the non-canonical pyroptosis pathway [41]. However, the coordinated upregulation of NLRP3, GSDMD, and cleaved caspase-1 strongly suggests that the canonical pathway is the predominant mechanism in this experimental system. NLRP3 inflammasome activation, due to its potent amplification effect on inflammatory cascades, typically overshadows the contribution of non-canonical pathways [42]. The expression of NLRP3 and GSDMD in the prefrontal cortex neurons of the HS6 rats was significantly increased, while the expression of NeuN remained unchanged. Since pyroptosis is an inflammatory form of programmed cell death characterized by cell membrane rupture, cell swelling, and the release of cellular contents, it may not lead to complete degradation of neuron-specific proteins, such as NeuN, in the early stages [43]. NeuN is located in both the nucleus and cytoplasm, and its loss of immunoreactivity may require a longer period of cellular disintegration. Therefore, the experimental time point likely captured the early stages of pyroptosis, during which NeuN could still be detected [44,45]. Therefore, despite detecting caspase-4 activity by ELISA, the molecular signature confirmed that the pyroptotic process was predominantly mediated through the caspase-1-dependent pathway. These findings reveal that HS-induced pyroptosis in the rat prefrontal cortex is primarily activated by the canonical pathway, and the NLRP3 inflammasome may play a pivotal role in this mechanism.

Hyperhomocysteinemia is known to exacerbate oxidative stress, promote inflammation, and contribute neurological dysfunction [46]. A clinical study reported that febrile seizures, a prevalent neurological disorder in children, are associated with significantly elevated serum Hcy levels, which positively correlate with inflammatory markers [12]. Our data revealed that Hcy levels were significantly increased in both the serum and prefrontal cortex of the HS6 rats. Concurrently, we observed marked downregulation of the Hcy metabolic enzymes MTHFR and CSE in the prefrontal cortex, suggesting that HS could disrupt Hcy metabolism. These results indicate that HS elevates Hcy levels, disrupting both transmethylation and transsulfuration metabolic pathways. Current clinical practice commonly employs B-complex vitamin therapy to reduce patients’ Hcy levels [47]. In our study, we successfully reduced Hcy levels in HS rats (both in the serum and the prefrontal cortex) through B-complex vitamin gavage. The results demonstrated that lowering Hcy levels decreased mortality in HS rats and reduced inflammatory responses and NLRP3-mediated pyroptosis in prefrontal cortical neurons. These findings suggest that Hcy contributes to HS-induced pyroptosis in prefrontal cortical neurons.

Accumulating evidence has identified NLRP3-mediated neuronal pyroptosis as a critical mechanism in neurodegenerative disorders such as Alzheimer’s disease [48]. Activation of the NLRP3 inflammasome triggers caspase-1-dependent pyroptosis, in which GSDMD forms pores in the plasma membrane, and activated caspase-1 processes pro-IL-1β and pro-IL-18 into mature cytokines, thereby amplifying the inflammatory responses [49]. Due to its broad pathogen recognition capacity, NLRP3 has emerged as a prime therapeutic target for modulating pyroptosis [50]. Our in vitro experiments on Hcy-induced pyroptosis showed elevated levels of LDH, IL-18, and IL-1β in the supernatants, alongside upregulated gene expression of AIM2, IL-6, NLRP1, and NLRP3. However, the gene expression of caspase-1 and GSDMD remained unchanged. The timing may be a key reason for the unchanged expression of caspase-1 and GSDMD in the Hcy-induced pyroptosis PC12 cell model [40]. The expression and activation of pyroptosis-related proteins are time-dependent. In the early stages, the primary observations may include the release of inflammatory factors and the upregulation of inflammation-related genes. However, significant changes in the expression of pyroptosis execution proteins may require a longer duration to become apparent [51]. MCC950 treatment effectively suppressed Hcy-induced pyroptosis by normalizing the expression of AIM2, IL-6, NLRP1, and NLRP3, reducing LDH and IL-18 release, and decreasing intracellular IL-1β levels. However, extracellular IL-1β levels showed minimal reduction. Potential explanations include the following: (1) MCC950 inhibits NLRP3 inflammasome assembly, limiting caspase-1 activation and the subsequent processing of pro-IL-1β [52,53]; (2) IL-1β secretion may lag behind production during sustained inflammation [54]; (3) alternative release pathways (e.g., microvesicles/lysosomes) may operate independently of caspase-1 [40]; and (4) IL-18 maturation strictly requires caspase-1, whereas IL-1β utilizes multiple inflammasomes and secretory mechanisms [39]. The results suggest that NLRP3 serves as a critical effector molecule in Hcy-mediated pyroptosis in prefrontal cortical neurons of HS rats.

N6-methyladenosine (m^6^A) stands out as one of the most common and widespread internal modifications found in eukaryotic mRNA and non-coding RNA. It is widely distributed across diverse species and tissues where it plays a crucial role in regulating gene expression [13]. There is evidence showing that m^6^A modification can mitigate neural damage, suppress inflammation, and reduce pyroptosis through distinct molecular mechanisms [16]. The methylation status of m^6^A is dynamically regulated by three classes of regulatory proteins: methyltransferases (“writers”), demethylases (“erasers”), and m^6^A-binding proteins (“readers”) [55]. As key executors of m^6^A signaling, the reader proteins include YTHDF1, YTHDF2, YTHDF3, YTHDC1, and YTHDC2. Among these, YTHDF2 specifically recognizes m^6^A sites through its YTH domain to regulate mRNA stability [56,57]. Hcy can be converted to cysteine via the transsulfuration pathway or remethylated to regenerate methionine [58]. Methionine is then transformed into S-adenosylmethionine (SAM), the primary methyl donor in cells, which participates in the methylation of DNA, RNA, and proteins [59]. According to the literature, the SAM/SAH ratio serves as an indicator of cellular transmethylation potential, with a reduction in this ratio signifying a diminished methylation capacity [60,61]. Elevated Hcy levels lead to increased accumulation of SAH, resulting in a reduction in the SAM/SAH ratio [62]. This study demonstrated that, at the organism level, HS induces an elevation in Hcy levels in the prefrontal cortex of rats, accompanied by a reduction in overall mRNA m^6^A and NLRP3 m^6^A methylation levels, as well as a decrease in YTHDF2 expression. Lowering Hcy levels was shown to alleviate HS-induced neuronal pyroptosis and the reduction in YTHDF2-mediated m^6^A methylation modification. Additionally, the in vitro cell experiments confirmed that elevated Hcy levels increased pyroptosis in PC12 cells, reduced the overall m^6^A and NLRP3 m^6^A methylation levels, and inhibited YTHDF2 expression. These findings suggest that YTHDF2 may have a potential regulatory role, possibly by modulating mRNA stability to influence the m^6^A methylation pattern, thereby inducing neuronal pyroptosis in HS rats. However, the precise molecular mechanisms need to be further investigated [63]. These results not only provide novel insights into the epigenetic regulation of HS-associated neuropathology but also identify the Hcy-m^6^A-YTHDF2-NLRP3 axis as a potential therapeutic target. Vitamin B supplementation demonstrates promising neuroprotective effects by breaking this pathogenic cascade at its metabolic origin.

## 5. Conclusions

In summary, HS induces prefrontal cortical damage in rats, which is characterized by increased neuronal pyroptosis that is predominantly mediated through the NLRP3-dependent classical pathway. Concurrently, HS triggers a significant elevation in Hcy levels in the prefrontal cortex, likely due to inducing metabolic disturbances. Our findings suggest that Hcy may contribute to HS-induced brain injury in rats by mediating neuronal pyroptosis via the m^6^A-YTHDF2-dependent NLRP3 pathway. The precise molecular mechanisms underlying this process warrant further investigation.

Our findings emphasize the critical importance of early therapeutic intervention during the initial window period following HS onset. The NLRP3 inflammasome and its downstream signaling pathways emerged as promising therapeutic targets for HS treatment. Future studies should further assess the clinical potential of targeting these mechanisms to develop more effective treatment strategies.

## Figures and Tables

**Table 1:** List of primers for qRT-PCR.

Gene	Primer Sequence (Forward)	Primer Sequence (Reverse)
*β-actin*	*CTTCCTGGGTATGGAATCCT*	*TCTTTACGGATGTCAACGTC*
*AIM2*	*AAATGCTGTTGTTGACCGGC*	*CTCCGTCCTGTCTGCAATGT*
*IL-6*	*ACAAGTCCGGAGAGGAGACT*	*TTCTGACAGTGCATCATCGC*
*NLRP1*	*GACCCTATGTGAGGTCCCCT*	*GGTGGTCTGTGAGGTCAGTG*
*NLRP3*	*TCTGTTCATTGGCTGCGGAT*	*TAGCCGCAAAGAACTCCTGG*
*GSDMD*	*CGACTCTGGAGAACTGGTG*	*TGGGTTTCACTCAACCCAG*
*caspase-1*	*GAAGATGATGGCATTAAGAAGG*	*CCAGGACACATTATCTGGTG*
*IL-18*	*TATCTGTGAAGGATGGAAGGA*	*TTTCAGGTGGATTCATTTCCTC*
*ASC*	*CACAATGACTGTGCTTAGAGAC*	*CACAGCTCCAGACTCTTCC*
*METTL3*	*ATGTGCAGCCCAACTGGATT*	*CTGTGCTTAAACCGGGCAAC*
*METTL14*	*TCCGGGAACGGCAGAAGTTA*	*CACAGCACCAATGCTATCTGC*
*WTAP*	*AGCAGCAACAGCAGGAATCT*	*GGTGCACTCTTGCATCTCCT*
*FTO*	*GGAGCGGGAAGCTAAGAAACT*	*GACCTCTTTGTGCAGCTCCT*
*ALKBH5*	*TGGCGCAAGTCCTATGAGTC*	*CTCATCTTCACCTTGCGGGT*
*YTHDF1*	*GGGGACAAGTGGTTCTCAGG*	*GCCTTGTTGAGGGTGTCACT*
*YTHDF2*	*AGTAGGGCAACAGACACAGC*	*AGTAGATCCAGAACCCGCCT*
*YTHDF3*	*ACTTTCAAGCACACCACCTCA*	*TGGCTTCCTCCTCCTCTTGA*
*YTHDC1*	*CAGCCGGGAGGAGAAAGATG*	*GAAGGCTTCTGTCGCTTGGT*
*YTHDC2*	*GCTCATGCAATGATGACCTGT*	*CCCGCTTGTCTTGCTCATTT*

## Data Availability

Data will be made available on request.
